# Value of reflectance confocal microscopy in guiding dermatological decision-making: insights from facial, body and oral lesion assessment

**DOI:** 10.1093/skinhd/vzag007

**Published:** 2026-03-31

**Authors:** Beatrice Bălăceanu-Gurău, Matteo Liberi, Francesco D’Oria, Giulio Foggi, Gisele Rezze, Marco Ardigò

**Affiliations:** Department of Oncologic Dermatology – ‘Elias’ Emergency University Hospital, ‘Carol Davila’ University of Medicine and Pharmacy, Bucharest, Romania; Dermatology Unit, IRCCS Humanitas Research Hospital, Rozzano, Milan, Italy; Department of Biomedical Sciences, Humanitas University, Pieve Emanuele, Milan, Italy; Dermatology Unit, IRCCS Humanitas Research Hospital, Rozzano, Milan, Italy; Department of Biomedical Sciences, Humanitas University, Pieve Emanuele, Milan, Italy; Dermatology Unit, IRCCS Humanitas Research Hospital, Rozzano, Milan, Italy; Department of Biomedical Sciences, Humanitas University, Pieve Emanuele, Milan, Italy; Dermatology Department, University of Trieste, Trieste, Italy; Dermatology Unit, IRCCS Humanitas Research Hospital, Rozzano, Milan, Italy; Department of Biomedical Sciences, Humanitas University, Pieve Emanuele, Milan, Italy

## Abstract

Our research letter highlights the utility of reflectance confocal microscopy (RCM) as a noninvasive, high-resolution imaging tool for evaluating cutaneous, mucosal and inflammatory lesions. By reducing unnecessary biopsies and improving diagnostic accuracy, RCM serves as a valuable adjunct to clinical and dermoscopic assessment, particularly in cosmetically sensitive areas.

Dear Editor, The pursuit of improved diagnostic accuracy in cutaneous tumours has driven the advancement of noninvasive techniques in dermatology, such as reflectance confocal microscopy (RCM). This imaging technique provides *in vivo*, cellular-resolution greyscale images of the epidermis, dermoepidermal junction and papillary dermis with near-histological detail, based on the refractive indices of the tissue.^1^ RCM helps reduce unnecessary biopsies and excisions, proving particularly valuable in cosmetically sensitive areas where preserving the skin’s appearance is essential.^1,2^ By enabling real-time, noninvasive assessment, RCM aids in accelerating the diagnostic process and optimizing the timing of therapeutic interventions, thereby enhancing clinical outcomes for patients.^1,2^

RCM is particularly valuable for distinguishing between benign and malignant lesions, both melanocytic and nonmelanocytic lesions, thereby guiding precise clinical management.^1,2^ It has proven especially effective in diagnosing and subtyping basal cell carcinoma through the recognition of its distinctive confocal characteristics, as well as in distinguishing precancerous lesions from squamous cell carcinoma, particularly in anatomically sensitive regions such as the lips.^3^ Furthermore, a randomized clinical trial demonstrated that adjunctive use of RCM for evaluating suspicious melanocytic lesions significantly reduces unnecessary excisions while ensuring timely removal of aggressive melanomas.^4^

Beyond oncological applications, RCM offers substantial benefits in the evaluation of inflammatory dermatoses.^5^ It enables the visualization of microscopic features with high interobserver reproducibility and a strong correlation to conventional histology.^5^ It has been shown to assist in diagnosing inflammatory and autoimmune skin conditions, grouped histologically into psoriasiform, spongiotic, bullous disorders, interface dermatitis and scleroderma.^1^ Valuable work by Ardigò *et al*. supports the feasibility of using RCM as a prehistological diagnostic tool for inflammatory oral mucosal diseases, highlighting its expanding role beyond traditional skin lesions.^6^

Over a 10-week period, we collected 61 cutaneous lesions referred for RCM evaluation at the Dermatology Unit, IRCCS Humanitas Research Hospital, Rozzano, Milan, Italy. This letter reflects a short-term, real-world observational experience from our institution, summarizing RCM referrals and subsequent management decisions over a 3-month period. The study was not designed as a diagnostic accuracy analysis, as histopathological confirmation was obtained only for lesions that proceeded to excision based on clinical and RCM findings. Although previous studies suggest that RCM is more commonly employed for facial lesions than those located on the body, we observed no significant difference in referral rates between these anatomical sites, with 33 (54%) facial lesions and 28 (46%) on other body sites.^7^ Most facial lesion referrals were based on clinical suspicion of lentigo maligna or had been scheduled for surgical excision. RCM evaluation revealed eight actinic keratoses (13%), seven basal cell carcinomas (11%), seven solar lentigo (11%), three lichen planus-like keratoses (LPLK; 5%), three melanocytic naevi (5%), two lentigo maligna (3%), two cases of sebaceous hyperplasia (3%) and one squamous cell carcinoma (2%).

Among the body lesions, most were suspected to be atypical naevi or melanomas and were also referred for excision. RCM assessment identified 10 congenital naevi (16%), 9 atypical naevi (15%), 3 basal cell carcinomas (5%), 2 seborrhoeic keratoses (3%) and 1 LPLK (2%), along with 3 inflammatory conditions, namely contact dermatitis, rosacea and lichen planus (5%). In our experience, 10 facial lesions (7 basal cell carcinomas, 2 lentigo maligna and 1 squamous cell carcinoma) and 3 body lesions (3 basal cell carcinomas) required surgical excision. Of the 61 lesions, 13 (21%) were excised, while the remaining patients were managed conservatively with follow-up recommended. Our findings demonstrate how RCM can support more conservative management by reducing unnecessary excisions, particularly in real-world settings where clinical, dermoscopic and confocal features are integrated into decision-making.

Three additional oral mucosal lesions, two cases of melanosis (67%) and one case of Bowen disease involving the salivary gland (33%), were evaluated separately due to their distinct anatomical, histological and diagnostic characteristics; accordingly, they were not included in the overall analysis or in Table 1 in order to preserve methodological consistency. It was proposed that the patient with Bowen disease involving the salivary gland was treated with topical imiquimod in light of its location and the patient’s preference, whereas the patients with melanosis were managed conservatively.

**Table 1:** Distribution of diagnoses based on reflectance confocal microscopy evaluation of facial and body lesions (*n* = 61)

Lesion type	Facial lesions	Nonfacial lesions
Basal cell carcinoma	7 (11)	3 (5)
Actinic keratosis	8 (13)	
Lentigo maligna	2 (3)	
Squamous cell carcinoma	1 (2)	
Solar lentigo	7 (11)	
Congenital naevus		10 (16)
Atypical naevus		9 (15)
LPLK	3 (5)	1 (2)
Melanocytic naevus	3 (5)	
Sebaceous hyperplasia	2 (3)	
Seborrhoeic keratosis		2 (3)
Inflammatory lesions		3 (5)

Data are presented as *n* (%). LPLK, lichen planus-like keratosis.

A limitation of our study is the absence of histological confirmation or structured follow-up for nonexcised lesions, as most patients were managed conservatively. As our observations were limited to a 3-month period, long-term outcomes of clinically monitored lesions remain unknown. In addition, the small number of observations for each lesion type limits the ability to reliably calculate diagnostic performance parameters such as sensitivity, specificity, positive predictive value, negative predictive value and overall accuracy. Nevertheless, clinical management was guided by the combined use of RCM, dermoscopy and clinical evaluation, in line with real-world practice. Furthermore, our findings are consistent with previous large-scale studies that have validated the diagnostic accuracy of RCM. Future studies involving larger, multicentre cohorts, with integrated correlations to histopathology and a more detailed assessment of practical considerations such as patient selection criteria, accessibility, cost and operator expertise would further clarify the real-world role of RCM. Additionally, combining RCM with complementary modalities such as dermoscopy and artificial intelligence–based decision-support tools is a promising direction to enhance diagnostic accuracy and standardize clinical workflows.

Facial lesions are considered a diagnostic challenge due to overlapping, site-specific clinical and dermoscopic features that reflect the unique histomorphology of facial skin, along with the limitations of biopsy in this cosmetically and functionally sensitive area.^8^ Ahlgrimm-Siess *et al*. demonstrated that the number of benign lesions on the face that were incorrectly diagnosed as malignant and destined for biopsy was reduced by 30%.^8^ Pellacani *et al*. proved that RCM decreases unnecessary excisions by analysing a consecutive sample of 3165 suspected lesions across the entire body.^4^

In addition to RCM, line-field confocal optical coherence tomography has recently shown promise for the evaluation of facial lesions, offering high-resolution three-dimensional imaging, and may serve as a complementary tool in noninvasive dermatological diagnostics.^9^

Our findings highlight the feasibility and clinical value of RCM as a noninvasive, prehistological diagnostic modality for cutaneous neoplasms, inflammatory dermatoses and oral mucosal lesions. Performing as a third-level examination, RCM complements clinical and dermoscopic assessments by providing real-time, *in vivo* microscopic imaging that enhances diagnostic accuracy and guides clinical management. Its utility in minimizing unnecessary biopsies is particularly advantageous in cosmetically and anatomically sensitive areas.

## Author contributions

Beatrice Bălăceanu-Gurău (Conceptualization [equal], Data curation [equal], Formal analysis [equal], Investigation [equal], Methodology [equal], Resources [equal], Software [equal], Supervision [equal], Validation [equal], Visualization [equal], Writing—original draft [lead], Writing—review & editing [equal]), Matteo Liberi (Conceptualization [equal], Data curation [equal], Formal analysis [equal], Investigation [equal], Methodology [equal], Resources [equal], Software [equal], Validation [equal], Visualization [equal], Writing—original draft [equal], Writing—review & editing [equal]), Francesco D’Oria (Conceptualization [equal], Data curation [equal], Formal analysis [equal], Investigation [equal], Methodology [equal], Resources [equal], Software [equal], Validation [equal], Visualization [equal], Writing—original draft [equal], Writing—review & editing [equal]), Giulio Foggi (Conceptualization [equal], Data curation [equal], Formal analysis [equal], Investigation [equal], Methodology [equal], Resources [equal], Software [equal], Validation [equal], Visualization [equal], Writing—original draft [equal], Writing—review & editing [equal]), Gisele Rezze (Conceptualization [equal], Data curation [equal], Formal analysis [equal], Investigation [equal], Methodology [equal], Project administration [equal], Resources [equal], Software [equal], Validation [equal], Visualization [equal], Writing—original draft [equal], Writing—review & editing [equal]), and Marco Ardigò (Conceptualization [lead], Data curation [equal], Formal analysis [equal], Funding acquisition [lead], Investigation [equal], Methodology [equal], Project administration [lead], Resources [equal], Software [equal], Supervision [lead], Validation [lead], Visualization [lead], Writing—original draft [equal], Writing—review & editing [equal])
